# The influence of membrane bilayer thickness on KcsA channel activity

**DOI:** 10.1080/19336950.2019.1676367

**Published:** 2019-10-13

**Authors:** Karen M. Callahan, Benoit Mondou, Louis Sasseville, Jean-Louis Schwartz, Nazzareno D’Avanzo

**Affiliations:** a From the Département de pharmacologie et physiologie, Faculté de médecine, Université de Montréal, Montréal, Canada; b Département de biochimie et médecine moléculaire, Université de Montréal, Montréal, Canada; c Centre SÈVE, Université de Sherbrooke, Sherbrooke, Canada

**Keywords:** Hydrophobic thickness, potassium channel, activation, inactivation, open probability, unitary current, single channel current

## Abstract

Atomic resolution structures have provided significant insight into the gating and permeation mechanisms of various ion channels, including potassium channels. However, ion channels may also be regulated by numerous factors, including the physiochemical properties of the membrane in which they are embedded. For example, the matching of the bilayer’s hydrophobic region to the hydrophobic external surface of the ion channel is thought to minimize the energetic penalty needed to solvate hydrophobic residues or exposed lipid tails. To understand the molecular basis of such regulation by hydrophobic matching requires examining channels in the presence of the lipid membrane. Here we examine the role of hydrophobic matching in regulating the activity of the model potassium channel, KcsA. ^86^Rb^+^ influx assays and single-channel recordings indicate that the non-inactivating E71A KcsA channel is most active in thin bilayers (<diC18:1PC). Bilayer thickness affects the open probability of KcsA and not its unitary conductance. Molecular dynamics simulations indicate that the bilayer can sufficiently modify its dimensions to accommodate KcsA channels without major perturbations in the protein helical packing within the nanosecond timescale. Based on experimental results and MD simulations, we present a model in which bilayer thickness influences the stability of the open and closed conformations of the intracellular gate of KcsA, with minimal impact on the stability of the selectivity filter of the non-inactivating mutant, E71A.

## Introduction

Atomic resolution structures of different potassium channels have provided valuable insights into the conformational changes that occur during opening, closing, and inactivation [1–9]. In particular, these crystal structures have identified a helix-bundle crossing gate formed by the cytoplasmic side of the TM2 (or S6) helices in the pore-domain, as well as a selectivity filter gate formed by the backbone carbonyls and a hydroxyl group the selectivity filter residues T(V/I)GYG. The helix-bundle crossing gate may be mechanically coupled to other regulatory domains, such as a voltage-sensor domain or a ligand binding domain, and is in many cases functionally coupled to the opening or closing of the selectivity filter gate [1]. However, the functions of many membrane proteins are regulated by the lipids in which they are embedded. These changes can arise from specific lipid–protein interactions or nonspecifically via the physiochemical properties of the bilayer. Nonspecific properties include surface charge, lateral stress, ordering of the lipid tails, and bilayer thickness. These regulatory mechanisms cannot be readily addressed by crystallographic approaches, even lipid-based methods, because of the heterogeneity and flexibility of the membrane bilayer, or its complete absence in detergent-based approaches. Hence, to gain dynamic information about the mechanisms of lipid regulation of membrane proteins, additional approaches are necessary.

The hydrophobic thickness of the bilayer has been shown to be an important regulator of several membrane proteins, including Ca^2+^-Mg^2+^ ATPase [10,11], Na^+^-K^+^ ATPase [12,13], diacylglycerol kinase [14], rhodopsin [15], nicotinic acetylcholine receptors [16] and calcium-activated potassium (BK_Ca_) channels [17,18]. This regulation in eukaryotic cells plays an important role in membrane sorting, as microdomains, organelles, and cells of different tissues vary in membrane thickness and adapt protein function to better suit the local sub-cellular requirements [19–21]. In humans, bilayer thickness can be altered through fatty acid intake, changes in cholesterol levels, and partitioning of small molecules. For example, partitioning of volatile anesthetics and drugs such as thiazolidinedione insulin sensitizers (TZDs) into cell membranes affect the function of numerous proteins embedded in them, including ion channels[22]. Matching of the bilayer hydrophobic thickness to the hydrophobic length of the membrane protein avoids large energetic penalties (3–260 kcal/mol for channel proteins[23]) due to the unfavorable solvation of hydrophobic residues in aqueous media. To accomplish this hydrophobic matching, protein helices in channel proteins can adjust their conformations by tilting, kinking, or stretching/compressing to match the thickness of the bulk bilayer, or alternatively, the bilayer can distort around the protein to become thicker or thinner.

KcsA is a pH-sensitive prokaryotic potassium channel found in the soil bacteria *Streptomyces lividans*[24], and has been extensively examined structurally [2,25,26] and functionally [27–33] as a model system for K^+^ channels. Wild-type (WT) KcsA inactivates within seconds following pH-dependent activation, whereas the mutation E71A, which resides adjacent to the selectivity filter, eliminates inactivation[32]. Thus, while WT KcsA has a low steady-state open probability, typically reported to be <0.1, E71A channels have an open probability approaching 1 at pH <4^28^. The function of KcsA channels also appears to be regulated by membrane lipids, with lipids required for refolding[34], and anionic lipids necessary for ion conduction through binding of the tetramer at residues on the extracellular side of the channel [34–37]. The strength of the fluorescence quenching of KcsA’s tryptophan residues by brominated lipids was observed to have a shallow dependence on the acyl tail length of bulk phosphatidylcholine (PC) lipids, which the authors suggest indicated that distortion of the KcsA channel is needed to achieve hydrophobic matching[36]. Additionally, acyl chain length of the bulk phospholipid inversely correlates with H^+^ affinity, and cooperativity[38]. However, the effect of bilayer thickness on ion permeation and gating in KcsA, and the molecular details of hydrophobic matching in these channels has yet to be addressed. In the present study, we show that the function of E71A KcsA channels is affected by changes in the bilayer thickness (driven by PC acyl chain length). Furthermore, MD simulations of both the closed and open states E71A KcsA channels in bilayers of different thicknesses provide qualitative insight into the contributions of protein adaptation and bilayer changes necessary to enable hydrophobic matching.

## Methods

### Purification of KcsA

E71A KcsA in the pQE32 vector were transformed into the *E. coli* strain BL21*pLysS. One liter of Luria-Bertani (LB) medium with 100 µg/mL Ampicillin was inoculated with a 25 mL pre-culture at 37°C until OD_600_ of 0.6, and then induced for 4 h with 1mM IPTG. Cells were harvested, resuspended in buffer (50 mM Tris-HCl, 150 mM KCl, pH 7) and lysed by freeze-thaw. Membranes were solubilized in 2% N-dodecyl-β-maltoside (DDM) for 2 h at 4°C. Solubilized membranes were clarified by centrifugation at 40,000g for 30 min and channels were bound to cobalt resin in batch mode for 2 h at 4°C. The resin was then packed into a gravity column, washed with 10 mM imidazole wash in a column and eluted with 500 mM imidazole. The eluted fractions were concentrated using a 30 MWCO spin concentrator, and polished by size exclusion chromatography.

### ^86^Rb^+^ influx assay

DiC_14:1_PC, diC_16:1_PC, diC_18:1_PC, diC_20:1_PC, diC_22:1_PC (phosphatidylcholine) and POPG (1-palmitoyl 2-oleoyl-phosphatidylglycerol) in chloroform were dried under nitrogen gas, and stored in a desiccator at −20°C. Lipids were solubilized in buffer A (150 mM KCl, 10 mM HEPES, 4 mM NMG pH 7.5) with 35 mM CHAPS at 10 mg/ml each, and mixed at 70 mol% PC and 30 mol% POPG. For each sample, 10–15 µg E71A KcsA protein was added to 100 µl of lipid (1 mg) and liposomes were formed by spinning the protein-lipid solution through a partially dehydrated column packed with Sephadex G-50 beads pre-equilibrated in buffer A at 1,000 x g (Beckman TJ6 centrifuge), and then spun through a second column, containing partially dehydrated beads pre-equilibrated in buffer B (150 mM sorbitol, 10 mM HEPES, 4 mM NMG, or pH 7.5). Uptake was initiated by adding 400 µl of buffer B with 1–5 µl of 1 µCi ^86^Rb^+^ (Perkin Elmer). For each experiment, a sample without protein was included as a measure of background ^86^Rb^+^ counts to be subtracted from all channel-mediated ^86^Rb^+^ uptake counts. At various time points, 60 µl aliquots were flowed through 0.5 ml Dowex cation exchange columns, mixed with scintillation fluid and counted on a liquid scintillation counter. All ^86^Rb^+^ uptake counts at 120 swere then normalized to counts of valinomycin-mediated uptake, a measure of maximum liposome capacity [39]. All experiments were performed at 20–22°C.

### Electrophysiology

Liposomes of 70 mol% diCnPC (n = 18:1, or 20:1) phosphatidylcholine and 30 mol% phosphatidylglycerol (POPG) were formed by sonication, and 20 μg of protein was added to the liposomes, mixed, and passed through a Sephadex G-50 spin column to remove the detergent. Proteoliposomes (<100 µL) were dialyzed against 14 mL of buffer A at 4°C for 12 h using a 10K MINI Dialysis device (ThermoFisher Scientific), with buffer exchanged after 6 h. Single channel recordings were collected from bilayers assembled in a vertical planar system with two chambers separated by a partition containing a 150–250 μm diameter hole. Bilayers of identical composition to the liposomes were formed manually by applying 10 mg/ml of 70 mol% diCnPC (n = 18:1, or 20:1) and 30 mol% POPG in decane over the hole in the partition. Bilayer formation was monitored electrically using a cycling voltage ramp. The *cis*-chamber contained (in mM): 90 KCl, 10 KOH, 10 Hepes at pH 7 adjusted using HCl. The *trans*-chamber contained (in mM): 90 KCl, 10 KOH, 10 succinic acid at pH 4 adjusted using succinic acid. Data were collected at 50 kHz using an Axopatch-1D amplifier with a lowpass filter of 2 KHz, digitized by a Digidata 1440A and recorded using Clampex software (Molecular Devices). All experiments were performed at 20–22°C. Unitary conductance and open probability analysis was performed in Clampfit and dwell time analysis was performed using QuB [40]. Further analysis and data plotting was performed in Origin8.0 (Microcal).

### Small angle x-ray scattering (SAXS)

Lipid bilayer thicknesses were measured at 20°C by small angle x-ray scattering (SAXS) using the F-2 beamline at MacCHESS synchrotron facilities (Cornell University, Ithaca, NY) at 9.881 keV. Liposomes of 70 mol% diCnPC (14:1–22:1) and 30 mol % POPG in buffer A pH 7.5 were generated by 49 passes through a 50nm PC membrane using a lipid extruder (Avanti Polar Lipids). A quantity of 200 µL samples were exposed 10 times for 30 s each and scattering data were collected using a Dual Pilatus 100K-S SAXS/WAXS detector. To avoid radiation damage, the samples were oscillated continuously throughout measurements. Intensity as a function of momentum transfer:

(1) $$q=4\pi \;sin\left(\theta \right)/\lambda$$

where λ is the wavelength of the beam, and θ is the angle of diffraction [41], was plotted and analyzed online using Bioxtas RAW v0.99.12b software [42]. Scattering from buffer A alone was subtracted from liposome samples to determine lipid dependent scattering. Electron density of the bilayer and parameters of the bilayer, including hydrophobic thickness (or interleaflet distance between the first carbon atoms of the lipid acyl tail following the glycerol backbone), were determined by a 3-Gaussian model derived from Lorentzian fits of the I-q relationship [43,44].

The Form Factor (Fh) is determined from the square root of the area under the Lorentzian curve fitting the I-q relationship determined by SAXS, and used to determine the electron density ρ*(z) of the lipid bilayer according to the relationship:

(2) $$\rho *\left(z\right)=\Sigma \pm F_{h}cos\left(qz\right)$$

where z is the distance of the system (d-spacing) in Å units. This expression is then converted to a tri-gaussian curve according to the following equation:

(3) $$\begin{array}{rl} & \rho \left(z\right)=exp\left[-{\left(z-z_{H}\right)}^{2}/2{\sigma_{H}}^{2}\right]\\ & \;\;\;\;\;\;\;\;\;\;\;\;+exp\left[-{\left(z+z_{H}\right)}^{2}/2{\sigma_{H}}^{2}\right]-\rho_{r}exp\left(-z^{2}/2{\sigma_{c}}^{2}\right) \end{array}$$

where z_H_ is the distance between the center of the bilayer and the lipid head-group, σ_H_ is the width of the gaussian describing the headgroups, ρ_r_ is the ratio between the electron density of acyl tails and the headgroup, and σ_C_ is the width of the Gaussian curve describing the hydrophobic core.

### Molecular dynamics

Systems for molecular dynamic simulations of open and closed states E71A KcsA were generated using CHARMM-GUI and the membrane builder [45–47]. The atomic structure for WT KcsA channel (PDB: 1R3J) was used to study the closed pore state of KcsA, since at the time of our calculations full-length structures were of lower resolution (~3.8 Å) [7,48,49]. The Tl^+^ in the selectivity filter was replaced with K^+^ and 36 pore waters were included. Pre-equilibrated protein coordinates of the 23 Å open state of KcsA (PDB: 3F7V)[1] with the selectivity filter in the active conformation were contributed by the Roux laboratory [50]. pH gating of KcsA on the intracellular side is attributed to a network of charged and titratable residues [31]. The pKas of amino acid residues were calculated using Propka 3.1 (Supp. Fig. S2) [51,52], and all glutamic acids and histidines present at the intracellular gate (E71, E118, E120, H25, H124) were protonated to represent an intracellular pH of 4. E71 was protonated in consideration of previous computational and experimental evidence [53–55]. The L90C mutation present in many crystal structures was reversed.

Three types of lipid binding sites have been described experimentally for KcsA. There are four non-annular binding sites between subunits on the extracellular side, and the intracellular and extracellular annular rings. The non-annular sites are occupied by anionic phospholipids like POPG[56], with binding in at least three sites required for channel opening [35,37]. The extracellular annular ring has no preference for zwitterionic lipids, like PCs, over anionic lipids[57]. The intracellular gate of KcsA contains many charged lipids and under pH 4, protonation of histidines and glutamates results in a net positive charge, thus the intracellular annular ring shows a preference for negatively charged lipids (POPG)[57]. The competition of lipids for these binding sites on KcsA cannot be sampled from our equilibrium simulations, which are limited to the nanosecond timescale. Therefore, to mimic the three types of lipid binding sites that have been described experimentally for KcsA (extracellular non-annular lipids, as well as the intracellular and extracellular annular rings), we recreated the protein-lipid solvation conditions to resemble those predicted by experiment as shown in Figure 3(a). Open and closed KcsA structures were embedded into a pure POPG membrane in 0.5 M KCl for 10 ns to allow POPG to briefly relax into the non-annular sites and form the intracellular annular ring. The protein, pore water and potassium, and POPG bound or adjacent to the non-annular sites and intracellular annular ring were embedded into a new a bilayer of diC_n:1_PC only lipids (n = 16–24). This second system underwent energy minimization, followed by 150 ps of dynamics to establish that there were no bad contacts. Then, extracellular annular ring composed solely of the PC lipid was selected. The finalized systems were composed of the protein, pore waters and ions, and the lipids in the non-annular binding sites and both annular rings. This was embedded into a membrane where each leaflet had a ratio of 30 mol% POPG and 70 mol% PC lipid, including the bound lipids. Occupation of the extracellular annular ring was chosen for comparability of solvation condition and to show the greatest possible effect of hydrophobic thickness on the activity of KcsA.

System sizes were approximately 145 Å x 145 Å x 90 Å. Systems were simulated in the NPT ensemble, at T = 303.15 K (30ºC) and p = 1 atm, using a 2 fs time step and keeping all bonds involving H atoms at constant length. Coordinates were recorded every 2 ps. The Charmm36 force field was used. This force field gives the correct area per lipid for neat membranes and POPG+POPC membranes when the NPT ensemble is employed [58,59] and is an improvement over Charmm27, which underestimates area per lipid when used in the NPT ensemble [60,61]. Standard CHARMM cutoff schemes and periodic boundary conditions were used: the Van der Waals non-bonded cutoff was 12 Å. Electrostatic interactions were calculated using particle mesh Ewald[62]. A Langevin piston was used for pressure control in a flexible cell with x-y coupling. And temperature was controlled with a Langevin thermostat. The protein was briefly equilibrated with RMSD restraints on the heavy atoms of the protein (k = 5 kcal/mol/Å^2^) incrementally released over ~1 ns to allow local solvent equilibration. A quantity of 200 ns of unrestrained MD was performed using NAMD 2.9 [63]. RMSD calculations over the course of the 200 ns trajectories of the final systems (Supp. Fig. S3) indicated each system had reached equilibrium. All analyses were performed on the final 150 ns of production simulations, although time-courses of several properties are reported for the continuous 200 ns trajectories in the supplement. The analysis and visualization codes were written using tcl within VMD [64], python, AWK, and gnuplot.

### Thin-layer chromatography

Liposomes of 70 mol% diCnPC (n = 16:1–22:1) phosphatidylcholine and 30 mol% phosphatidylglycerol (POPG) were formed as described for ^86^Rb^+^ influx assays and 3 µL of liposomes were spotted onto a silica coated K6F TLC plate (Whatman). TLC was run using a 65 CHCl_3_: 24 MeOH: 4 H_2_O solvent system and compared to lipid standards. Results were visualized by iodine staining. Densitometry measurements were performed using ImageJ software (NIH) and are reported in Supp. Fig. S1.

## Results

### KcsA function is regulated by membrane thickness

**Figure 1.**
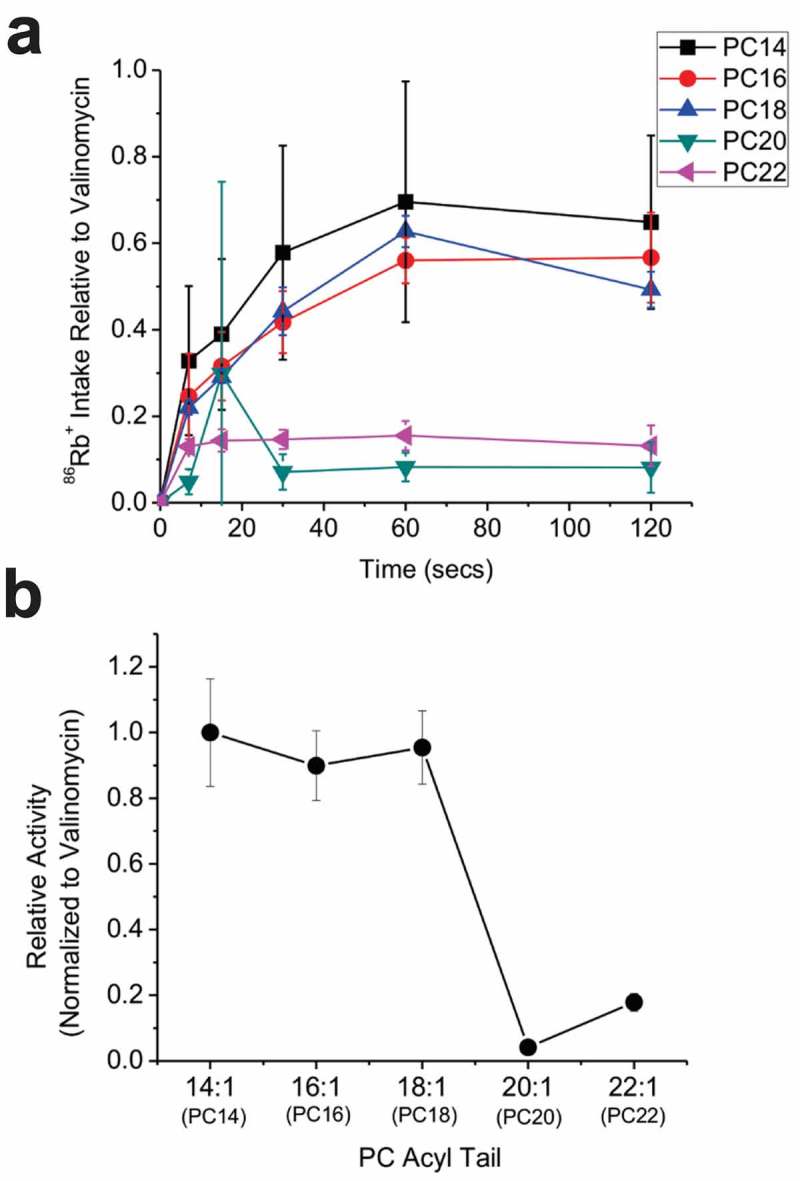
KcsA activity depends on membrane thickness. (a) Time courses of ^86^Rb^+^ influx into proteoliposomes containing 70 mol% of PC lipids varying in acyl tail length and 30 mol% POPG reconstituted with E71A KcsA channels (n = 6). Uptake was normalized to valinomycin, which quantifies the maximal uptake possible. (b) Valinomycin normalized counts at 120 swere plotted relative to counts in diC14:1PC/POPG, where maximal activity was observed.

Bilayer thickness has also been shown to affect the pH sensitivity of KcsA channels [38]. However, to determine if bilayer thickness also affects the gating properties or conductance properties independently of pH, the function of KcsA was assessed at constant pH by ^86^Rb^+^ influx into liposomes containing PC lipids of varying acyl chain length. Since function of this channel depends on the presence of anionic phospholipids [34–36], we maintained the POPG concentration at 30 mol% in each condition. We observed that KcsA mediated ^86^Rb^+^ influx of the non-inactivating E71A mutant channels is dependent on the length of the PC acyl chain with which the proteoliposomes were formed (Figure 1). The function of E71A KcsA increases as chain length decreased, with maximal ^86^Rb^+^ influx observed in liposomes containing acyl tail lengths with 18 or less carbons (<diC18:1 PC).

**Figure 2.**
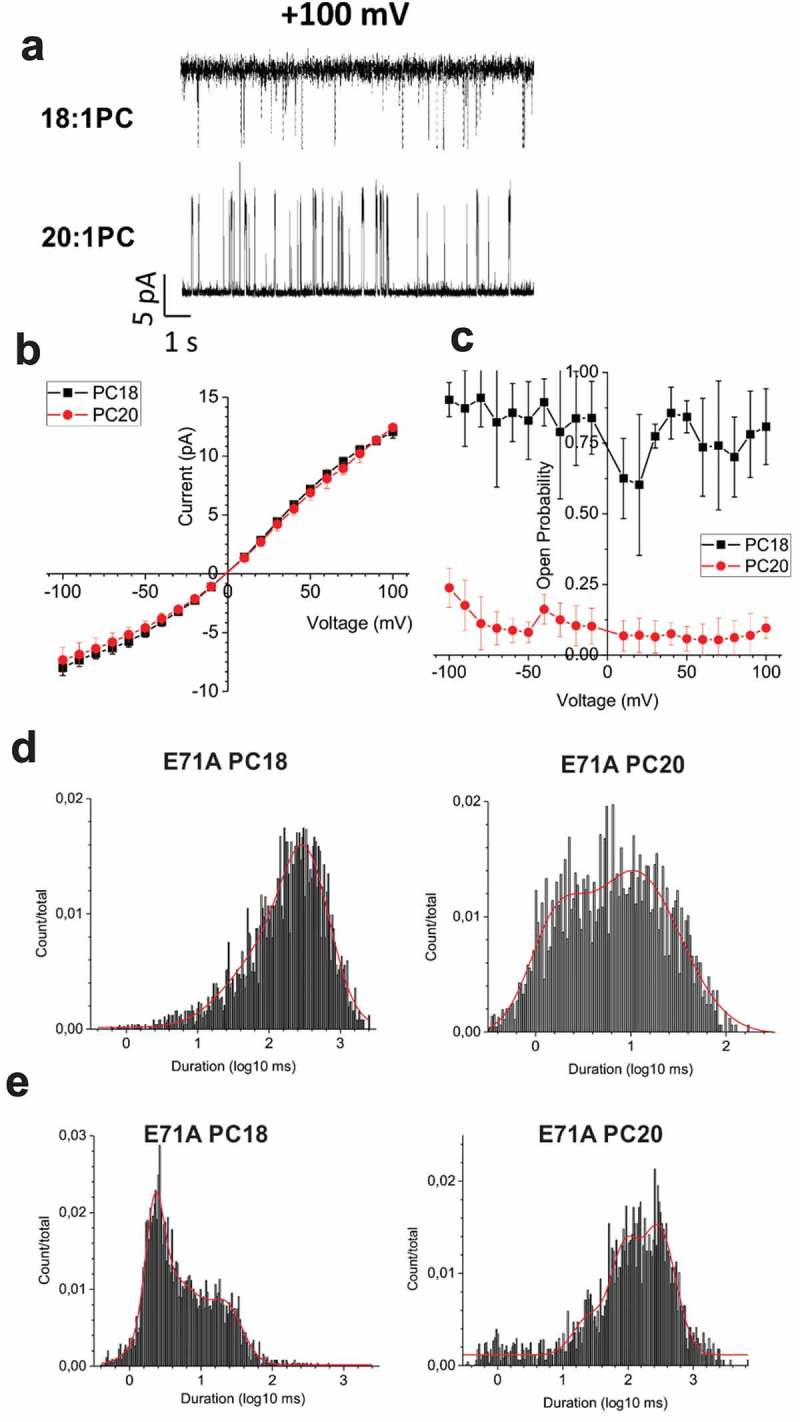
Single channel properties of E71A KcsA in lipid bilayers of different thickness. (a) Representative single channel traces at +100 mV E71A KcsA channels in bilayers composed of 30 mol% POPG and 70 mol% diC18:1PC (top) or diC20:1PC (bottom). (b) The unitary conductance of KcsA E71A channels are unchanged by variations in bulk lipid acyl tail length. (c) The open probability E71A is higher in diC18:1PC/POPG bilayers (right). (n ≥ 3). All-point histograms of open (d) and closed (e) dwell times of E71A channels at +100 mV in 30 mol% POPG + 70 mol% diC18:1PC or diC20:1PC.

To determine whether the bilayer thickness affects KcsA gating transitions (channel opening or closing) or unitary conductance we measured single-channel properties from E71A KcsA channels reconstituted into in planar lipid bilayers (Figure 2). For these experiments, E71A KcsA channels were reconstituted into liposomes of 30 mol% POPG and 70 mol% diC18:1 or diC20:1 and function was assessed of the bilayers of the same lipid ratio. Our recordings indicate that lipid acyl tail length does not affect the unitary current versus voltage relationship of either KcsA channels (Figure 2(b)). However, P_open_ of E71A KcsA was nearly 8 times larger in diC18:1PC (Popen ~0.8) than diC20:1PC (~0.1) at all voltages (Figure 2(c)). Consistent with previous studies [32,33], E71A KcsA shows no voltage dependence in either bilayer condition. Dwell time analysis of the single-channel recordings (Figure 2(d,e); Table 1) indicate both closed and open states are affected by changes in bilayer thickness. At −100 mV, E71A can enter into short (4 ms) and longer (110 ms) closed states in both in diC18:1 PC or diC20:1 PC lipids. However, an intermediate closed state of approximately 30 ms becomes apparent in longer lipids at this voltage. Similarly, at +100mV, the two closed states were prolonged by an order of magnitude, and a third 300 ms closed time was observable. On the other hand, E71A KcsA samples 1 or 2 prolonged open states in diC18:1 PC depending on the voltage. Intriguingly, these dwell times decrease by an order of magnitude in diC20:1 PC. Thus, long-chain lipids appear to stabilize several closed states and destabilize open states of E71A KcsA channels.

**Table 1.** Single channel dwell times for E71A KcsA in POPG + diC18:1 PC or diC20:1PC.

Voltage	PC18 closed time (ms)	PC20 closed time (ms)	PC18 open time (ms)	PC20 open time (ms)
−100 mV	4 ± 1	4 ± 2	1020 ± 537	5 ± 0
		32 ± 13		24 ± 5
	110 ± 4	104 ± 0		118 ± 0
+100 mV	3 ± 1	27 ± 6	69 ± 30	2 ± 1
	15 ± 8	163 ± 23	413 ± 251	14 ± 5
		321 ± 0

### Relationship between acyl chain length and hydrophobic thickness of the membranes

**Figure 3.**
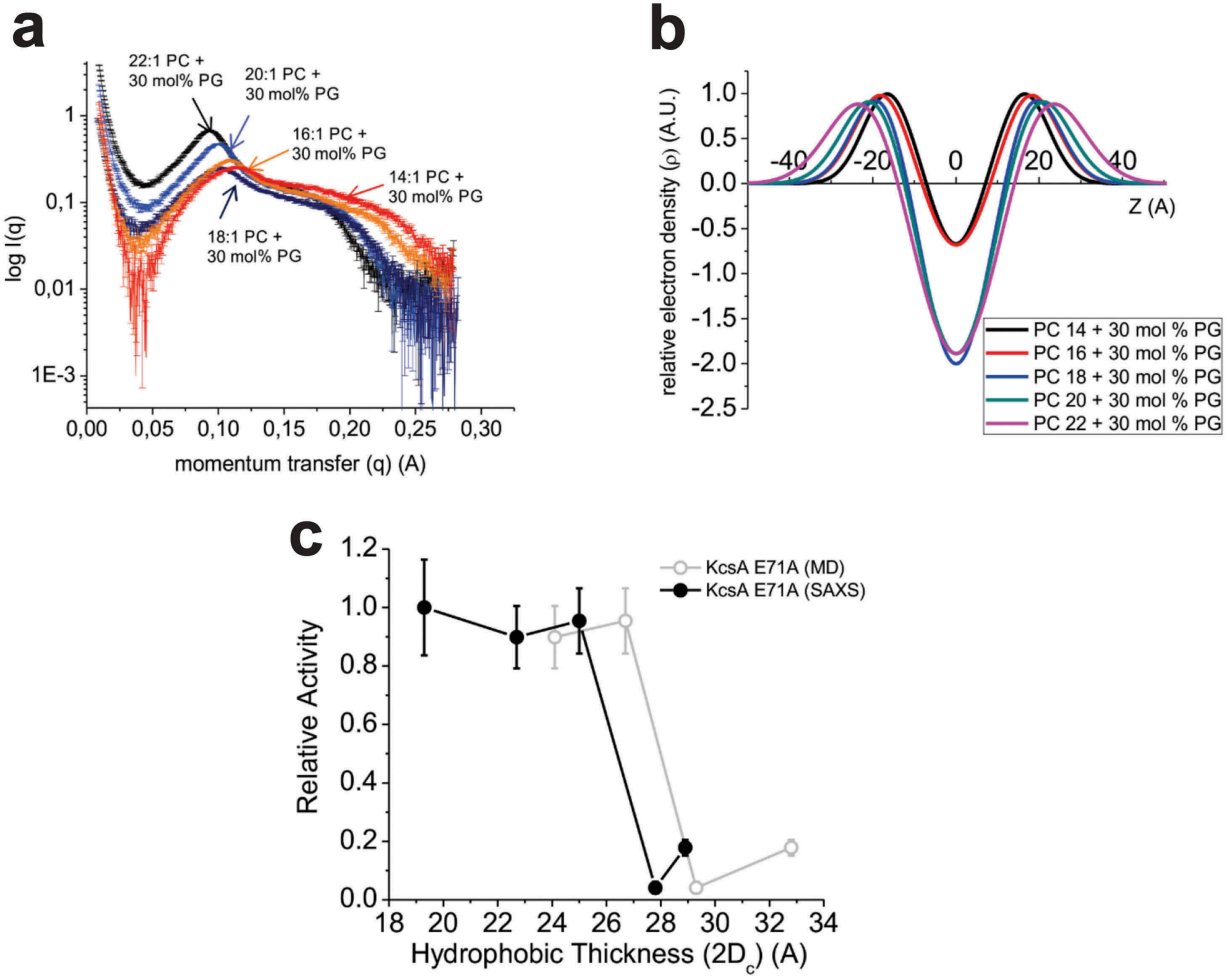
SAXS measurements of bilayer properties. (a) Intensity versus momentum transfer (q) plots of small-angle x-ray scattering (SAXS) data collected from 50 nm liposomes under the same experimental conditions used for single-channel and ^8^^6^Rb^+^ flux assays. (b) Electron density profiles calculated from SAXS data using a 3-Gaussian model. Specific parameters averaged from three experiments are characterized in Table 1. (c) The function of E71A KcsA channels measured by ^86^Rb^+^ flux was re-plotted against estimates of hydrophobic thickness from SAXS experiments (●) (Table 2) or from the average of the MD simulations (○) (Table 3). These data indicate that channel activity is modulated drastically with sensitivities in a range as narrow as 0.5–3 Å.

**Figure 4.**
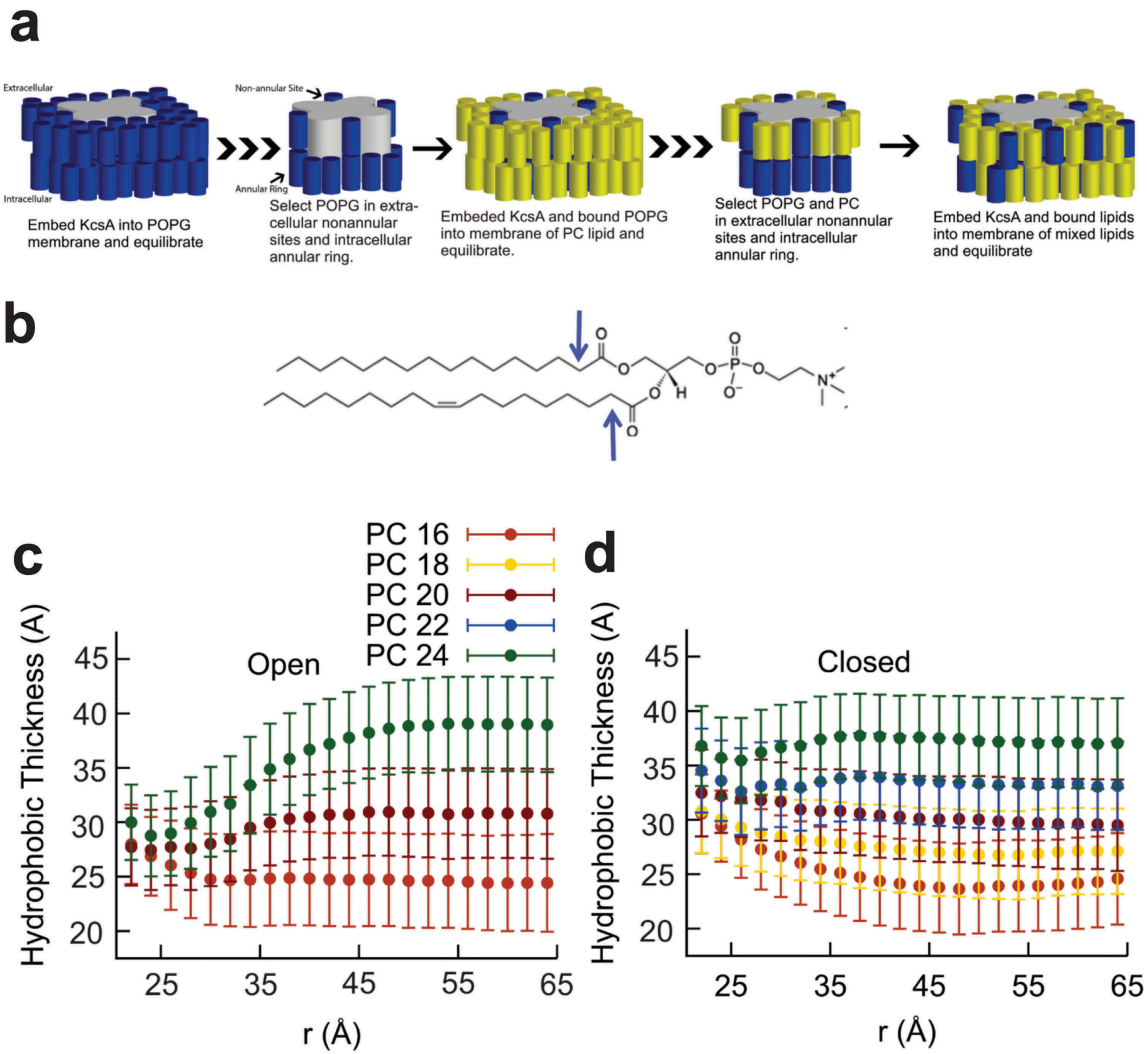
Molecular Dynamics simulations of E71A KcsA in various lipid membranes. (a) A cartoon describing the manner in which the MD simulations systems were built. KcsA channels were first embedded in a POPG only membrane and equilibrated. Lipids bound to the non-annular site and intracellular ring were selected, and the KcsA-POPG complex was then embedded into a PC only bilayer and equilibrated. The new KcsA-lipid bound complex was then selected and embedded into a 70 mol% PC 30 mol% POPG membrane that accounted for the number and types of lipids bound to the channel. (b) The first alkyl carbons of POPG and PC lipids (as highlighted by arrows for POPC) were used to determine the lipid hydrophobic thickness in a series of simulations of E71A KcsA channels in the open (c) and closed (d) states as a function of radial distance from the pore axis. Standard deviations were calculated using the method of blocking transforms.

To determine the relationship between the length of the lipid acyl chain and hydrophobic thickness, we performed small angle x-ray scattering (SAXS) experiments (Figure 3). Scattering intensity versus momentum transfer (I-q) (Figure 3(a)) was converted to an electron density profile using a 3-Gaussian model [43,44] (Figure 3(b)) and the bilayer properties including bilayer thickness were assessed (Table 2). Hydrophobic thickness increased 1–3 Å per additional 2 carbons on the acyl chain, with the increase being in the larger end of the range for bilayers with shorter lipid chains. Hydrophobic thickness of the lipid membrane was also determined molecular dynamics simulations of membrane only systems (Table 3). Specifically, we determined hydrophobic thickness from the radially averaged density of the first alkyl carbon of each chain after the backbone glycerol (highlighted with an arrow in Figure 4(b)) the average values >45 Å away from the protein axis. The estimates of bilayer thickness by SAXS and MD calculations (Tables 2 & 3) are within 4 Å of one another and are not deemed statistically significantly different. Moreover, the regular increase in hydrophobic thickness of bilayers with increasing chain length is reproduced by simulation. This allows us to comment on trends in relationships between hydrophobic thickness and protein behavior, even if MD bilayers are slightly thicker.

**Table 2.** Bilayer thickness parameters determined by SAXS.

Lipid mixture (+30 mol% PG)	Acyl chain (n_c_)	d-space (A)	Z_H_	σ_h_ (FWHM)	d_c_ (Z_H –_ σ_h_)	Hydrophobic thickness (2d_c_)	Bilayer thickness (d_b_)	Water layer (d_w_)	Head to head distance (d_hh_)
PC14	14	64.9 ± 1.5	16.0 ± 1.7	12.7 ± 1.4	9.7 ± 1.4	19.3 ± 2.0	44.7 ± 4.8	20.2 ± 6.3	32.0 ± 3.4
PC16	16	62.9 ± 0.6	18.0 ± 0.9	13.3 ± 1.6	11.3 ± 0.1	22.7 ± 0.2	49.3 ± 3.5	13.6 ± 2.9	36.0 ± 1.8
PC18	18	65.9 ± 1.4	20.2 ± 1.8	15.4 ± 2.4	12.5 ± 0.6	25.0 ± 1.1	55.9 ± 6.0	10.0 ± 4.6	40.4 ± 3.5
PC20	20	67.9 ± 0.3	21.2 ± 0.3	14.5 ± 0.5	13.9 ± 0.1	27.8 ± 0.1	56.9 ± 1.1	11.0 ± 0.9	42.3 ± 0.6
PC22	22	72.0 ± 0.3	21.4 ± 1.0	14.0 ± 1.0	14.4 ± 0.5	28.9 ± 1.0	56.9 ± 3.0	15.1 ± 2.7	42.9 ± 2.0

**Table 3.** Bilayer hydrophobic thickness determined by molecular dynamics simulation of KcsA.

Lipid mixture (+ 30 mol% PG)	Acyl chain (n_c_)	Absence of protein	E71A open	E71A closed
PC16	16	25.2 ± 2.5	24.5 ± 4.4	24.1 ± 4.1
PC18	18			26.9 ± 3.9
PC20	20	30.5 ± 2.6	30.7 ± 4.0	29.1 ± 4.1
PC22	22			34.1 ± 3.8
PC24	24	35.8 ± 2.8	38.7 ± 4.5	38.2 ± 3.8

### Hydrophobic matching between KcsA and lipid bilayer does not require large-scale conformational changes in the protein

Matching of the bilayer hydrophobic thickness to the hydrophobic length of the membrane protein avoids large energetic penalties due to the unfavorable solvation of hydrophobic residues in aqueous media. It has been suggested that hydrophobic matching can occur through conformational changes in the membrane protein (e.g. by tilting, kinking, or stretching/compressing of helices to match the thickness of the bulk bilayer), or alternatively, the bilayer can distort around the protein to become thicker or thinner. We therefore sought to determine the structural basis for hydrophobic matching and dissect the contributions of protein movements versus bilayer deformations. We performed all-atom molecular dynamics simulations on E71A KcsA channels in closed and open conformations embedded in bilayers containing 30 mol% POPG and 70 mol% PC of different tail lengths (Figure 4(a)). RMSD calculations of the transmembrane helices over the course of the 200 ns trajectories (Supp. Fig. 3) indicate the systems are fully equilibrated. Hydrophobic thickness of the lipid membrane was determined from the radially averaged density of the first alkyl carbon of each chain after the backbone glycerol (highlighted with an arrow in Figure 4(b)) in the upper and lower leaflet as a function of distance from the central pore axis of the protein. It is clear from these calculations that the membrane deforms in the region near the edge of the protein (r ~ 20 Å) for KcsA channels in both open and closed conformations, and that the hydrophobic thickness of the membrane near the protein is thinner when the protein is in the open state (Figure 3(c,d)). Thus, our simulations indicate that rapid expansion or contraction of the bilayer near the protein is sufficient to accommodate hydrophobic mismatch with KcsA channels and that this matching occurs on the timescale of nanoseconds.

**Figure 5.**
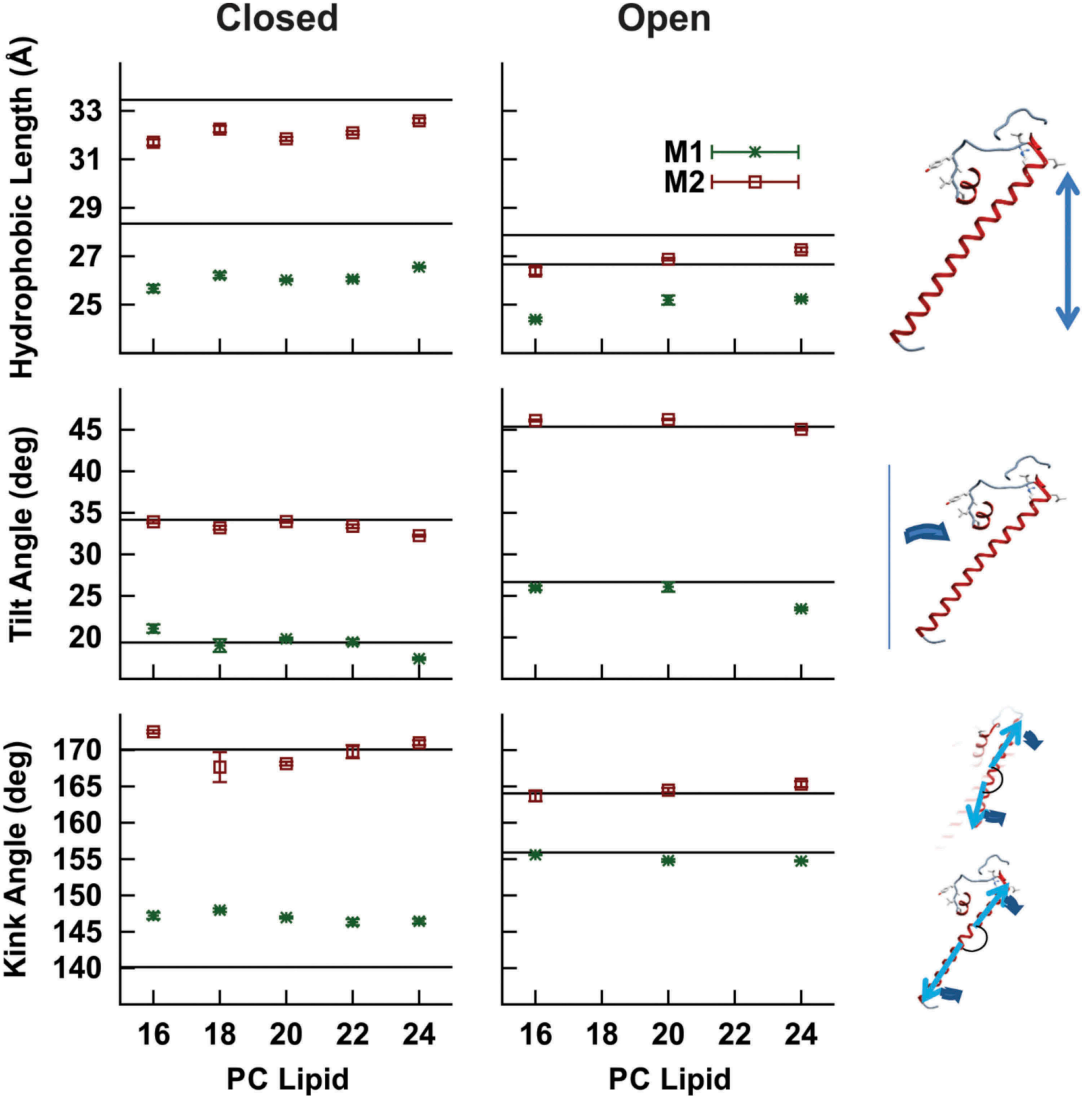
Time-averaged structural properties E71A KcsA helices in a series of lipid bilayers estimated from MD simulations. (a) The hydrophobic length of the M1 and M2 helices were calculated from the projection of the distance from residues 26 to 45 and 87 to 113, respectively, onto the normal of the bilayer for each lipid condition. (b) The angle of tilt away from the central pore axis was determined for the M1 and M2 helices of both proteins. (c) The kink angle was measured between the upper (residues 44 & 51 and residues 86 & 103) and the lower (residues 24 & 42 and residues 105 & 121) portions of the M1 and M2 helices, respectively. For (a-c) error bars were determined from the method of blocking transforms. The predicted values from the crystal structures of WT closed (PDB: 1R3J) and open KcsA (PDB: 3F7V) used for these simulations is shown in as a solid line.

To assess the role of protein perturbations in hydrophobic matching, we examined our molecular dynamics simulations of E71A KcsA embedded in the series of membranes. Helical movements (Figure 5) were computed using the time averages with errors calculated using the blocking transforms method in order to remove the effects of time correlation. Time-series data of these properties for the M1 and M2 helices of the four subunits of the protein are shown in Supplemental Figures 5–7. Over the course of the simulations for E71A KcsA channels, the hydrophobic length of these helices (distance of the residues L24 to E51 for M1 and L86 to R122 for M2 projected to the z-axis) changed in thickness by less than 1 Å as the carbon chains of the PC lipid were increased from a 16 to 24 (Figure 5(a)). This was the case in both the open and closed conformations for these proteins.

It has been previously suggested that the helices of KcsA channels may tilt away from the central pore axis significantly in order to accommodate hydrophobic mismatch[36]. Therefore, we calculated the helical tilt angles from the vectors leading from residue L24 to residue E51 in M1 and residue L86 and residue R122 in M2 (Figure 5(b)). In the crystal structure of closed WT KcsA, the M1 helix is tilted 20° from the central pore axis, while M2 has a tilt of approximately 33° (Figure 5(b); horizontal black lines). Both the M1 and M2 helices of E71A closed are similar to that of the crystal structure. In the initial open KcsA configuration, the tilt angles of the M1 and M2 helices are ~27° and 45°, respectively, which went unchanged over the course of our simulations.

The tilt angle alone may underestimate the extent of the conformational change in the protein structure, because it does not reveal changes in the helices due to bowing or kinking. Therefore, we examined the kink angle between the top and bottom of the M1 and M2 helices (Figure 5(c), and time-series data in Supp. Fig. 7). The kink angles of M1 and M2 of the WT closed crystal structure (PDB: 1R3J) were 140° and 169°, respectively. For the M2 helices, the kink angles are similar to that of the crystal structures (169°-173°), but for the M1 helices, the kink lessens compared to that in the crystal structure, giving a time-averaged value between 145° and 150°. These changes observed in kink angle are consistent with straightening of the helices in these lipid conditions, compared to the crystal structures, but did not differ between lipid conditions. Simulations of the open structure of KcsA did not undergo any changes in M1 and M2 kink with changes thickness of the lipid bilayer. These data are all consistent with the conclusion that hydrophobic matching in KcsA is primarily driven by rapid distortion of the lipid bilayer rather than large perturbations in the protein, and that large conformational perturbations must occur on longer timescales such as those probed by electrophysiological studies.

### Sensitivity to membrane thickness is not driven by changes at the selectivity filter

**Figure 6.**
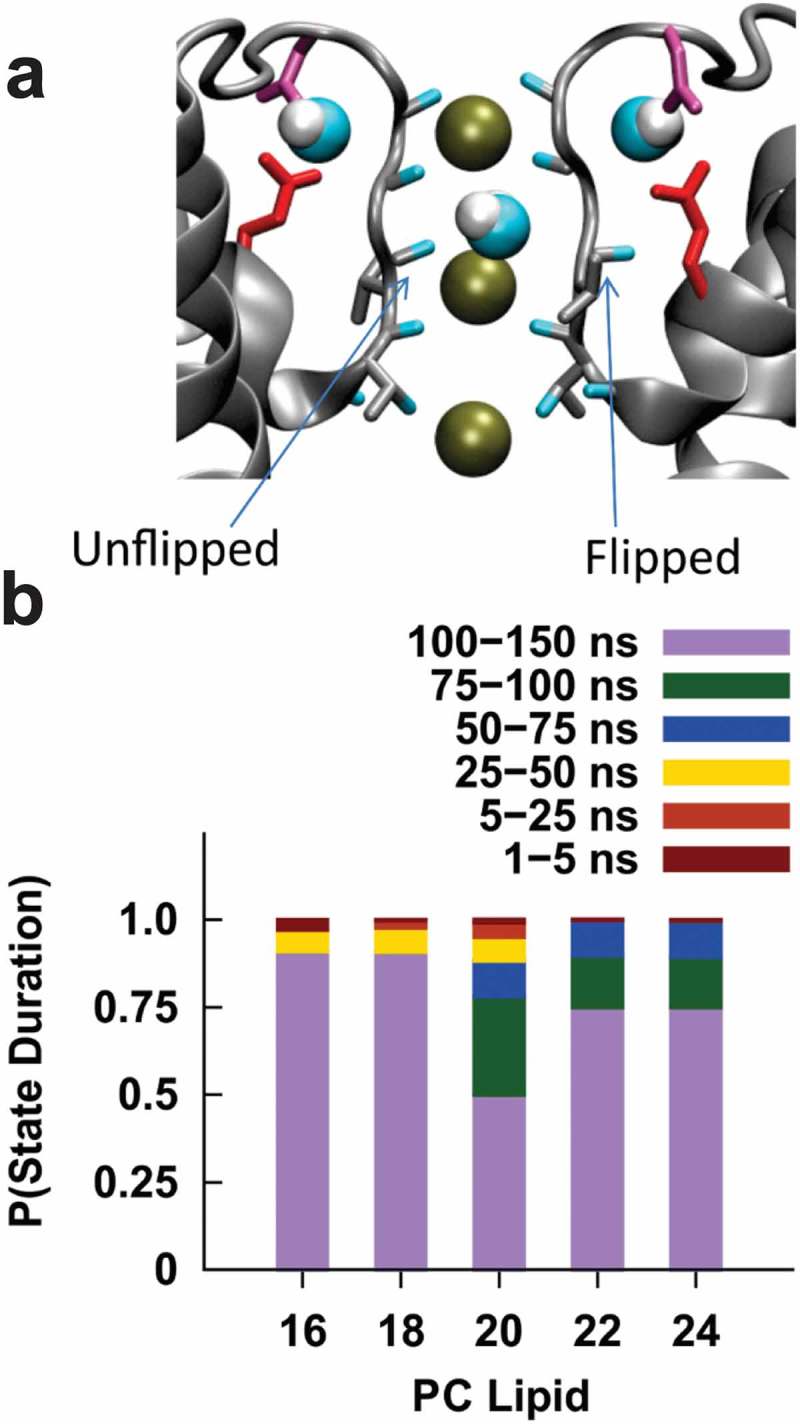
**Valine 76 carbonyl orientation in the selectivity filter E71A KcsA. (a)** The KcsA selectivity filter two chains removed clarity. The left chain has the backbone of V76 in the unflipped state, while the backbone of the right chain shows the flipped conformation. **(b)** Duration of V76 occupation in a flipped or unflipped conformation according to PC lipid composition. We observe that V76 in E71A KcsA channels are not very mobile, with long lived states (>75 ns) making up more than 75% of the transitions observed over the course of our simulations in all lipid conditions. These data suggest that bilayer thickness does not regulate E71A KcsA function by destabilizing the selectivity filter. Therefore, the effects at the TM2 gate are more prevalent in E71A.

The carbonyl groups lining the selectivity filter of KcsA have been observed to flip away from the pore axis leading to a non-conductive state[65] and may be linked to C-type inactivation [66]. This carbonyl flipping remains in the presence of the E71A mutation [66,67]. To determine if changes in membrane thickness (for example through changes in lateral tension) could alter the pore-stability, we assessed the amount of carbonyl flipping of the pore-lining residues (Figure 6). Time-series data for (the carbonyl groups of) T75, V76, and ^67^G77 are presented in Supplemental Figures 8–9. We observe that T75 remains facing the selectivity filter throughout the course of all our simulations (Supp. Fig. S9A). Flipping of V76 is common in simulations of E71A (Figure 6(b) & Supp. Fig. 9). As evident in the time-series data (Supp. Fig. 8B), in the closed state in E71A transitions between states are considerably rare and long-lived (Figure 6(b) & Supp. Fig. S8B). We did not identify a discernible lipid dependence to the flipping of residue G77 (Supp. Fig. S8C). We note that unlike in the closed state, in the open state the occasional conduction of ions meant the number of potassium in the pore was variable and not controlled or sufficiently sampled in the present study to draw conclusions about the selectivity filter of the open state. However, our data from the closed state simulations still indicate that the selectivity filter of E71A is stable, and that flipping of selectivity filter residues is largely unchanged in bilayers of different thicknesses. These results suggest that lipid thickness influences the stability of the open and closed conformations of the intracellular gate of KcsA rather than the selectivity filter. These data are consistent with the lack of changes we observe in E71A KcsA unitary conductance in bilayers of different thickness (Figure 2(a))[33].

## Discussion

We observed that the function of E71A KcsA channels is dependent on the acyl tail length of PC lipids that form the bulk of our experimental membranes. It does not seem as though the effect of membrane thickness is due to the favorable redistribution of the anionic phospholipids necessary for their function [34–36], since this mode of regulation would be expected to affect the unitary conductance[35]. This is corroborated by a recent study which indicated that the pH sensitivity and cooperativity of KcsA channel is sensitive to bilayer thickness and that these changes were not dependent on PG content of the bilayers[38]. Bilayer thickness has also been shown to affect the pH sensitivity of KcsA channels[38], however, our flux assays and electrophysiological experiments, as well as our simulations, were performed under conditions (intracellular pH = 4 or pH >7) in which the pH sensitivities of these channels were observed to plateau. Thus, our observations cannot be derived from differences in proton affinity. Taken together, these data indicate that membrane thicknesses can regulate the open to closed equilibria in KcsA channels. We therefore sought to understand how hydrophobic matching between the lipid bilayer and the hydrophobic length of the channel can occur.

Hydrophobic matching in KcsA channels has been initially examined using the sensitivity of fluorescence quenching in KcsA channels embedded in brominated phospholipid bilayers of differing chain length [36]. The interpretation of the small changes in the lipid binding constant with increasing chain length was taken to suggest protein distortions were necessary to match the lipid bilayer, and that the lipid did not distort to match the protein. Based on these data, it was predicted that to match bilayers of diC18:1PC, diC16:1PC and diC14:1PC, the tilt angle of the protein helices would have to adjust from an approximately 25° angle in the crystal structure to 43°, 50°, and 56°, respectively, [36]. To dissect the respective contributions of bilayer adaptation and protein conformational adjustments, we performed molecular dynamic simulations in bilayers of the same lipid composition as used in our experimental conditions. To ensure that our MD simulations were consistent with experimental conditions, we validated the hydrophobic thickness calculated beyond 40 Å from the protein using SAXS. Estimates of bilayer thickness by SAXS and MD calculations (Table 4) are within 4 Å, a difference that was not statistically significant. This is comparable to the differences observed in other lipid systems examined by MD and SAXS [68]. These differences may arise from the high electron density of anionic phospholipid head-groups which induces broadening of the peak in the Intensity – momentum transfer (q) SAXS profile (Figure 3) and may result in an underestimation of the hydrophobic thickness by this method. It is important to note that increasing KCl concentration up to 3M does not alter the electron densities of lipid bilayers[69], and thus any differences in potassium concentration between our experiments would be expected to have no effect on bilayer thickness. Our MD calculations of open and closed conformations of E71A show directly that the lipid membrane will readily distort to fit the hydrophobic region of the protein (Figure 4). On the other hand, gross protein conformational changes were minimal and not lipid dependent (Figure 5). Thus, on the nanosecond time-scale, hydrophobic matching in KcsA is nearly exclusively driven by membrane distortions, and not gross conformational changes of the channel helices. However, an ambulatory protein may then relax preferentially into the state or states that put less strain on the bilayer over a longer trajectory. An alternative interpretation of the fluorescence data, also supported by our single-channel analysis, is that these fluorescence changes report on changes in the distribution of discrete closed or open states, where the distribution is influenced by bilayer thickness, rather than progressive conformational changes[38].

**Figure 7.**
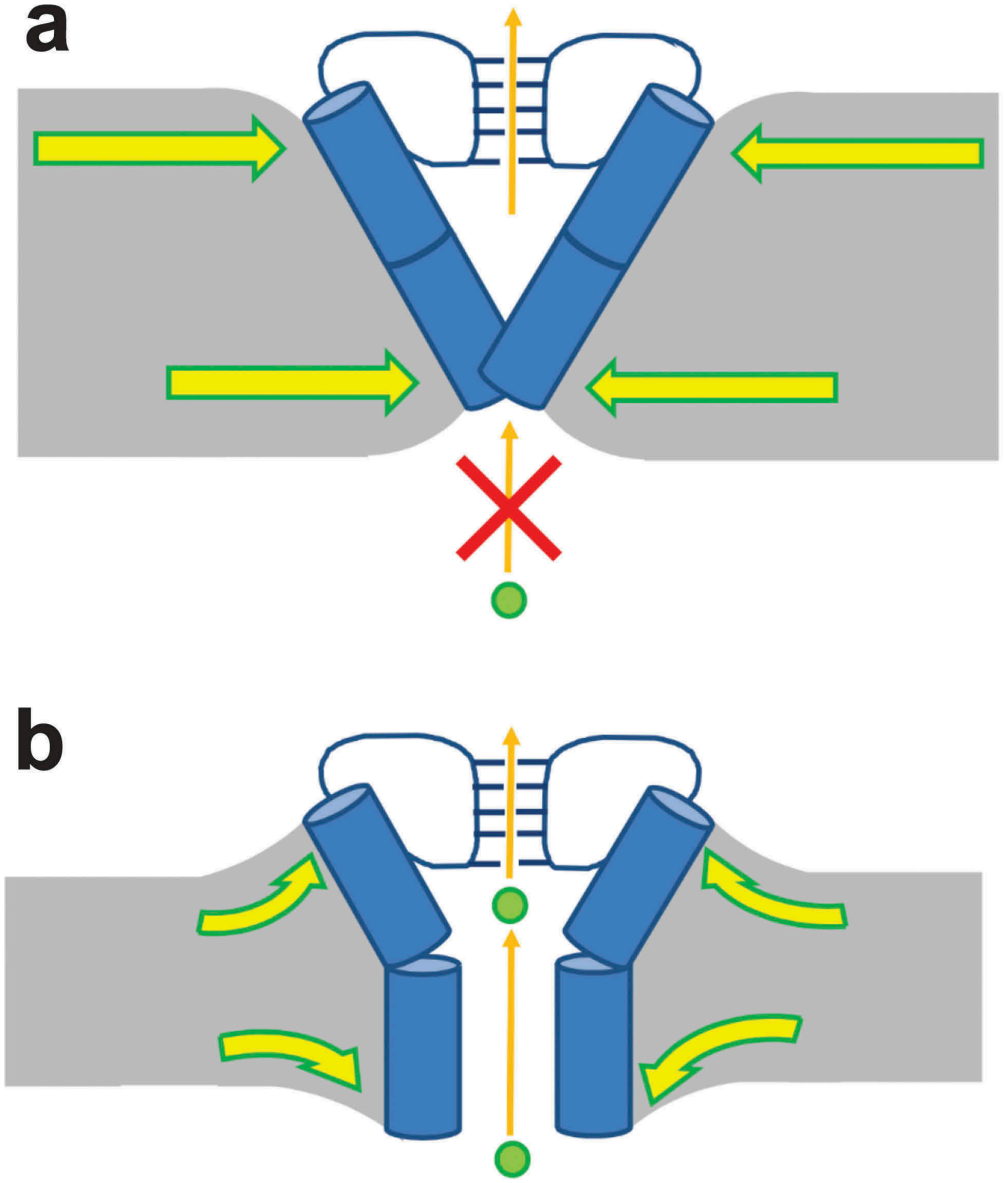
Model of hydrophobic thickness regulation of KcsA channel gating. (a) In thick bilayers, the membrane leaflets distort to accommodate hydrophobic mismatch. Lateral pressure favors closing of the TM2 gate. (b) In thin bilayers, the membrane leaflets distort to accommodate hydrophobic mismatch. The reduced lateral pressure enables TM2 gate to open more readily.

Our single channel analysis in planar lipid bilayers indicates that bilayer thickness regulates the open probability and not the unitary conductance of KcsA channels (Figure 2). As E71A channels lack C-type inactivation, these data suggest that the closed to open transition of KcsA channels is favored in thinner bilayers. While the timescale of our MD simulations is too short to observe large movements at the TM2 gate, the lack of large differences in the mobility of the selectivity filter residues suggests that bilayer thickness likely has the greatest impact on the TM2 gate rather than the pore gate. Therefore, to explain our data, we have developed a model of regulation of KcsA gating by bilayer thickness (Figure 7). We speculate that since lateral pressure in the bilayer increases with increasing thickness [70,71], opening of the TM2 gate is inhibited by the increasing lateral stress forces that the lipid molecules apply on the protein. Similarly, increased bilayer thickness was also proposed to increase the activation energy for opening of BK_Ca_^17^ and MscL channels[71]. Our data suggest that this intracellular gating is affected by bilayer thickness similarly in KcsA, with shorter lipids encouraging opening of this gate.

Cell membranes are dynamic systems with lipid profiles within the bilayers altering with time and cellular conditions. *Streptomyces lividans* are capable of synthesizing hopanoids, functional analogous of cholesterol in bacterial membranes[72], which increase lipid order [72] and thus membrane thickness [73,74]. This may have an effect on KcsA channel function, for example, during spore formation [75]. More broadly, changes in hydrophobic thickness of the bilayer appear to be a general modulator of potassium channels, as observed for BK_Ca_ channels BK_Ca_^17^ and other Kv channels [76–79]. This may contribute to fine-tuned control of these channels in tissue such as the heart and brain whose lipids generally differ in chain length or even sub-cellular domains such as cholesterol and sphingolipid-rich lipid rafts. Furthermore, membrane thickness modulation may also contribute to the actions of external K^+^ channel modulators such as anesthetics, C70 fullerene [79], or membrane-thinning toxins such as VSTx1 [77,78] and HaTx[76].
