# Supplementary material for: The influence of membrane bilayer thickness on KcsA channel activity
Source: Channels (Austin). 2019 Oct 13;13(1):424–39. doi: 10.1080/19336950.2019.1676367 (PMC6802934; doi:10.1080/19336950.2019.1676367)
Supplement: Supplemental Material [file kchl-13-01-1676367-s001.docx]

The influence of membrane bilayer thickness on KcsA channel activity

Karen M. Callahan^1*^, Benoit Mondou^2,*^, Louis Sasseville^1^, Jean-Louis Schwartz^1,2,3^, Nazzareno D'Avanzo^1,2^

^1^ From the Département de pharmacologie et physiologie, Faculté de médecine, Université de Montréal, Montréal, QC, Quebec;

^2^ Département de biochimie et médecine moléculaire, Université de Montréal;

^3^ Centre SÈVE, Université de Sherbrooke, Sherbrooke, QC, Canada;

***** These authors contributed equally

To whom correspondence should be addressed: Dr. Nazzareno D’Avanzo Telephone: (514) 343-5634, FAX: (514) 343-7146, e-mail: nazzareno.d.avanzo@umontreal.ca

**
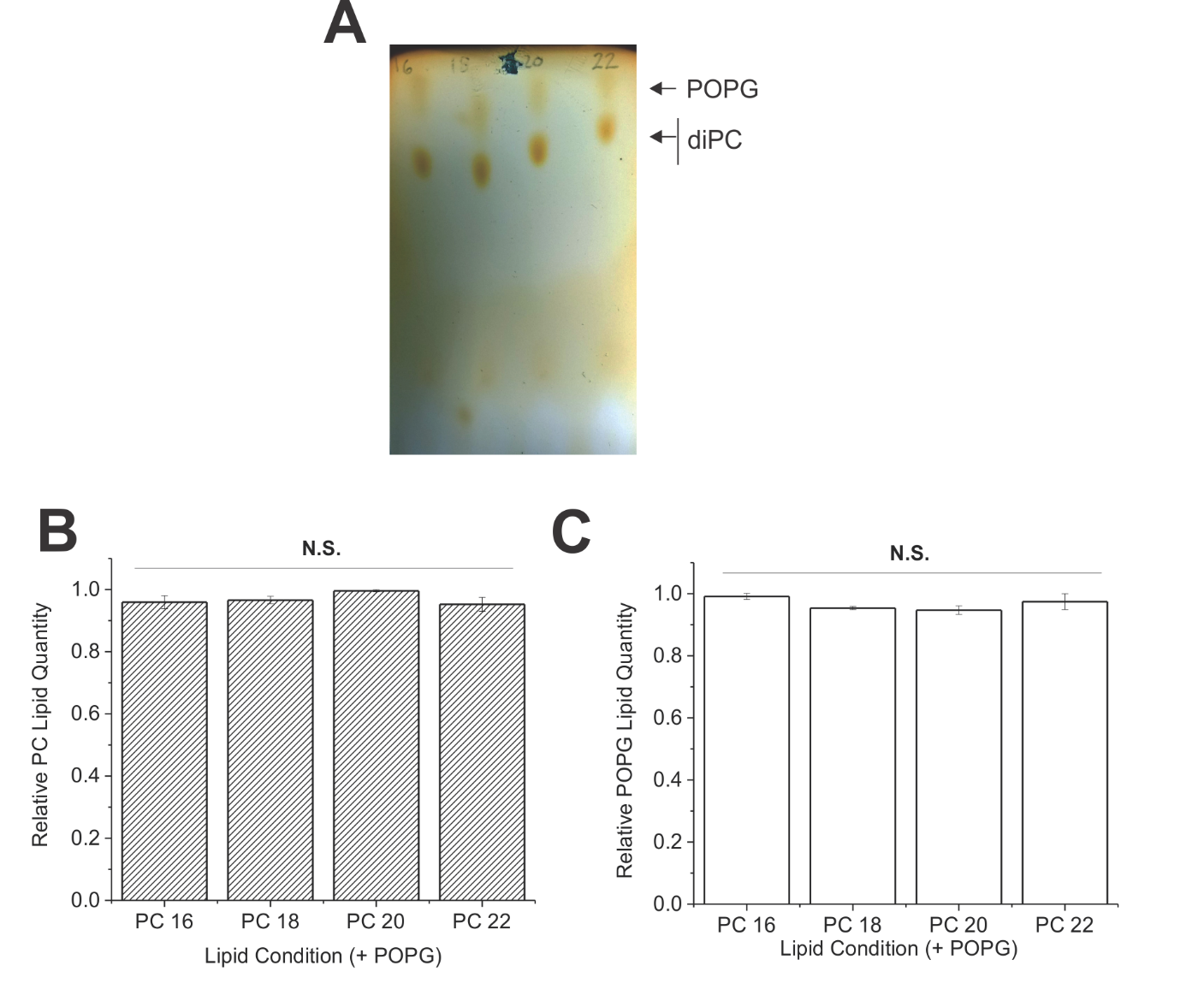
**

Supplemental Figure S1. Thin-layer Chromatography. (A) TLC used to assess any differences in the quantity of diPC and POPG lipids that form liposomes following the spin-column gel filtration method. Quantification of PC (B) or POPG (C) lipids by densitometry indicate no statistically significant difference between lipid conditions.

Supplemental Figure S2. Cross-sectional view of the KcsA pore domain. The N- and C-termini and front and rear subunits have been removed for clarity. Protein backbone is represented by white ribbons. Selected side chains, as well as the backbone carbonyls of the selectivity filter are represented as tubes. Arginines are green, glutamatic acids are red, aspartatic acids are mauve, and histidines are brown. Potassium ions (thallium in the crystal structure) are represented with brown spheres, and structural water is represented by cyan and white spheres. The position of the lipid (30% POPG + 70% PC22, in this case) is light purple. PROPKA3 was used to calculate pKa’s of relevant amino acid side chains.

**
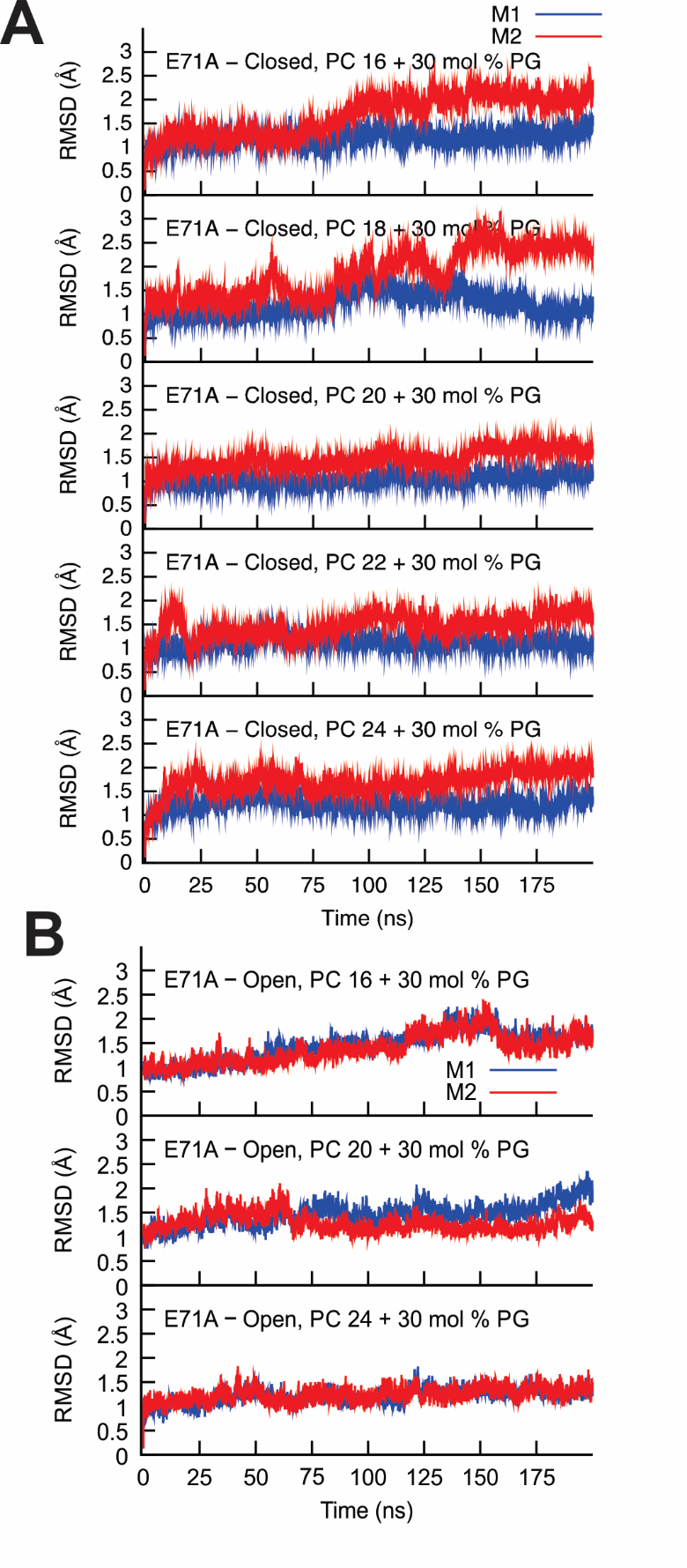
**

**Supplemental Figure S3. Root mean square deviation (rmsd) calculation** of the M1 (blue) and M2 (red) helices of WT and E71A KcsA with reference to the first frame of the simulation of the protein in the labeled bilayer.

**
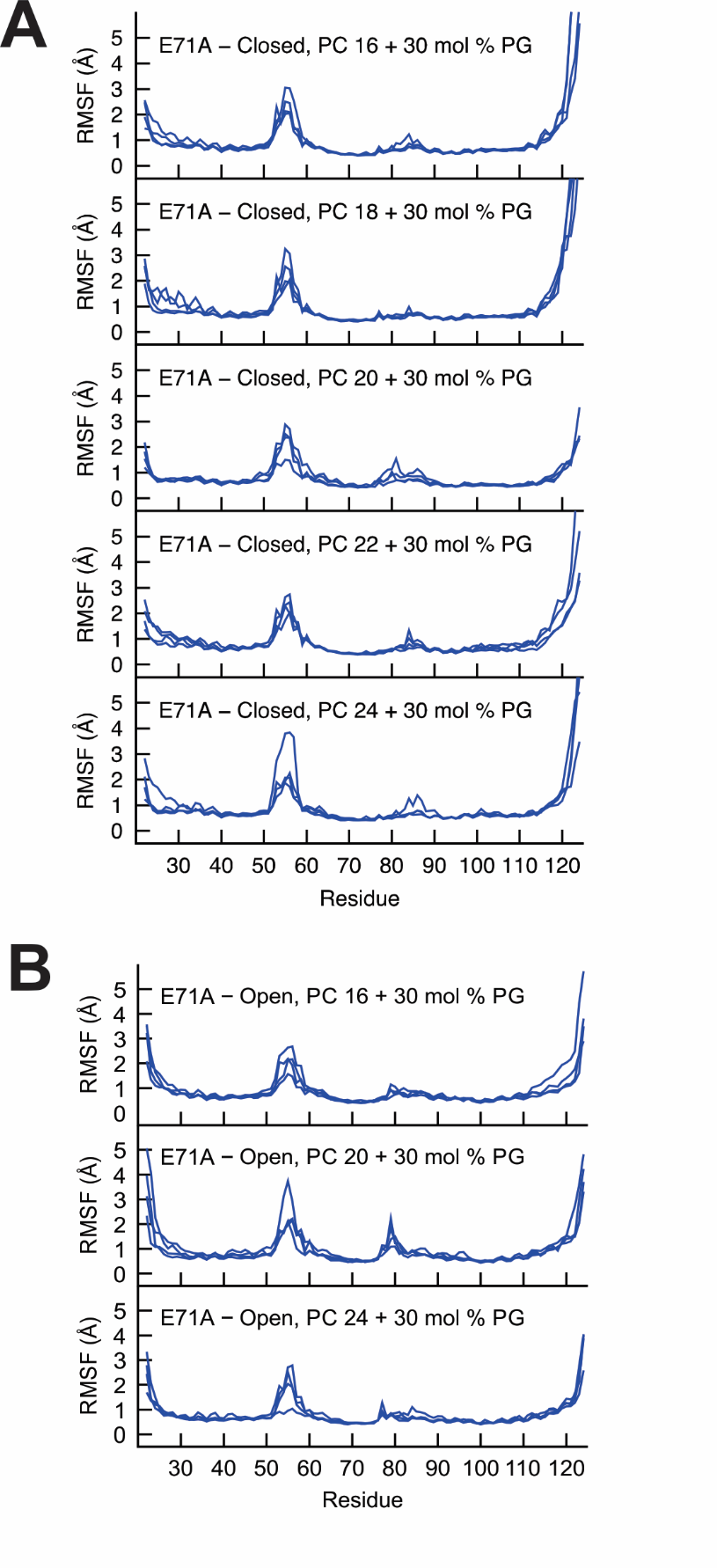
**

**Supplemental Figure S4. Root mean square fluctuations (rmsf)** of the protein in simulations of WT and E71A in a range of bilayer conditions. The result for each subunit is plotted as a separate line.

**
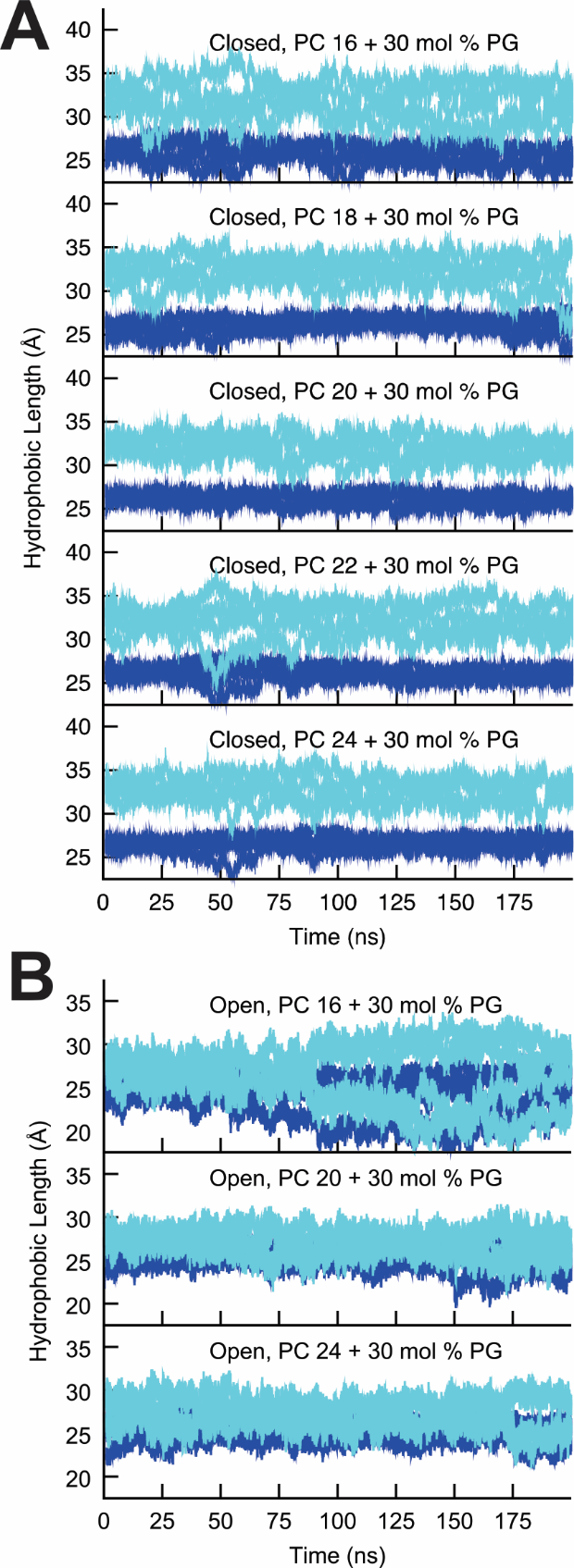
**

**Supplemental Figure S5**. Time-courses of hydrophobic length of the E71A KcsA helices in closed **(A)** and open **(B)** conformations in a series of lipid bilayers estimated from MD simulations. The hydrophobic length of the M1 and M2 helices were calculated from the projection of the distance from residues 26 to 45 and 87 to 113 respectively onto the normal of the bilayer for each lipid condition.

**
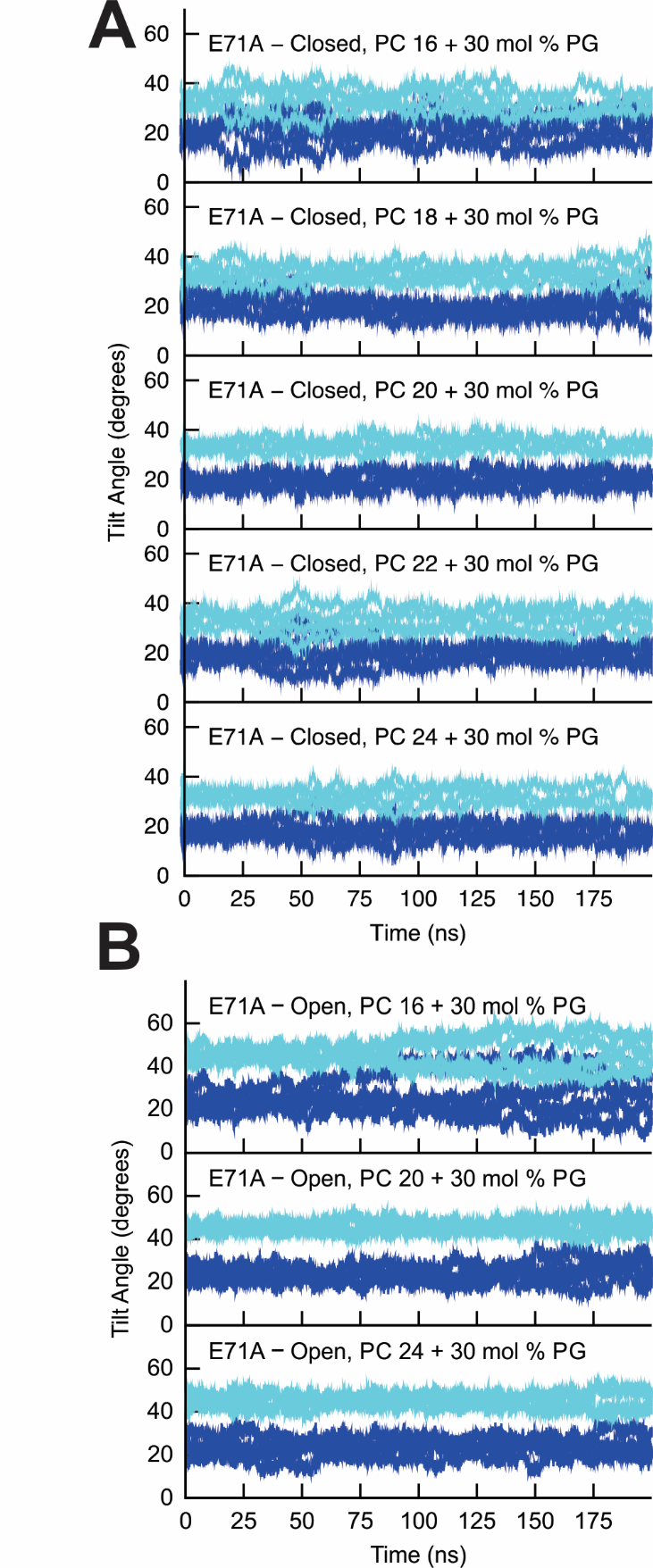
**

**Supplemental Figure S6.** Time-courses of helical tilt of the E71A KcsA helices in closed **(A)** and open **(B)** conformations in a series of lipid bilayers estimated from MD simulations. The angle of tilt away from the central pore axis was determined for the M1 and M2 helices of both proteins.

**
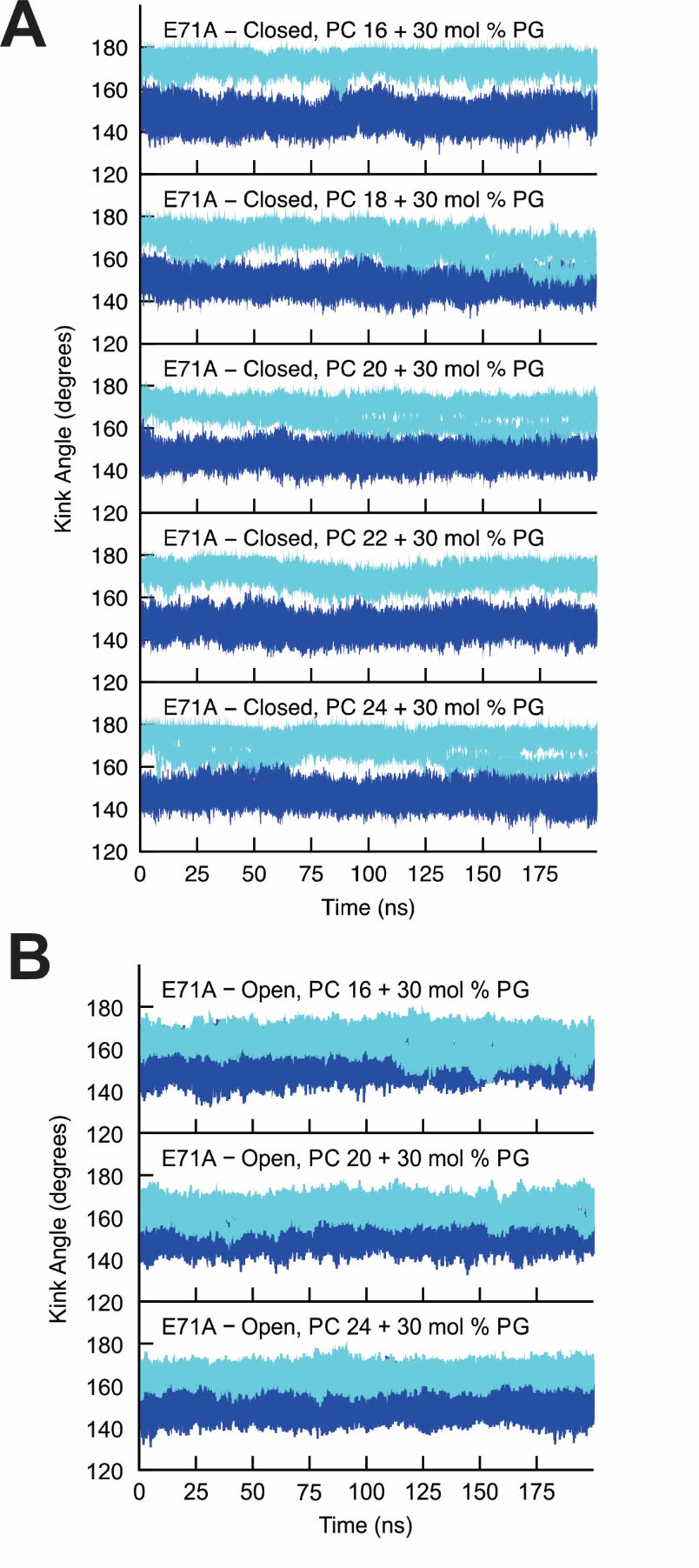
**

**Supplemental Figure S7.** Time-courses of kink angles of the E71A KcsA helices in closed **(A)** and open **(B)** conformations in a series of lipid bilayers estimated from MD simulations. The kink angle was measured between the upper (residues 44 & 51 and residues 86 & 103) and the lower (residues 24 & 42 and residues 105 & 121) portions of the M1 and M2 helices respectively.

**
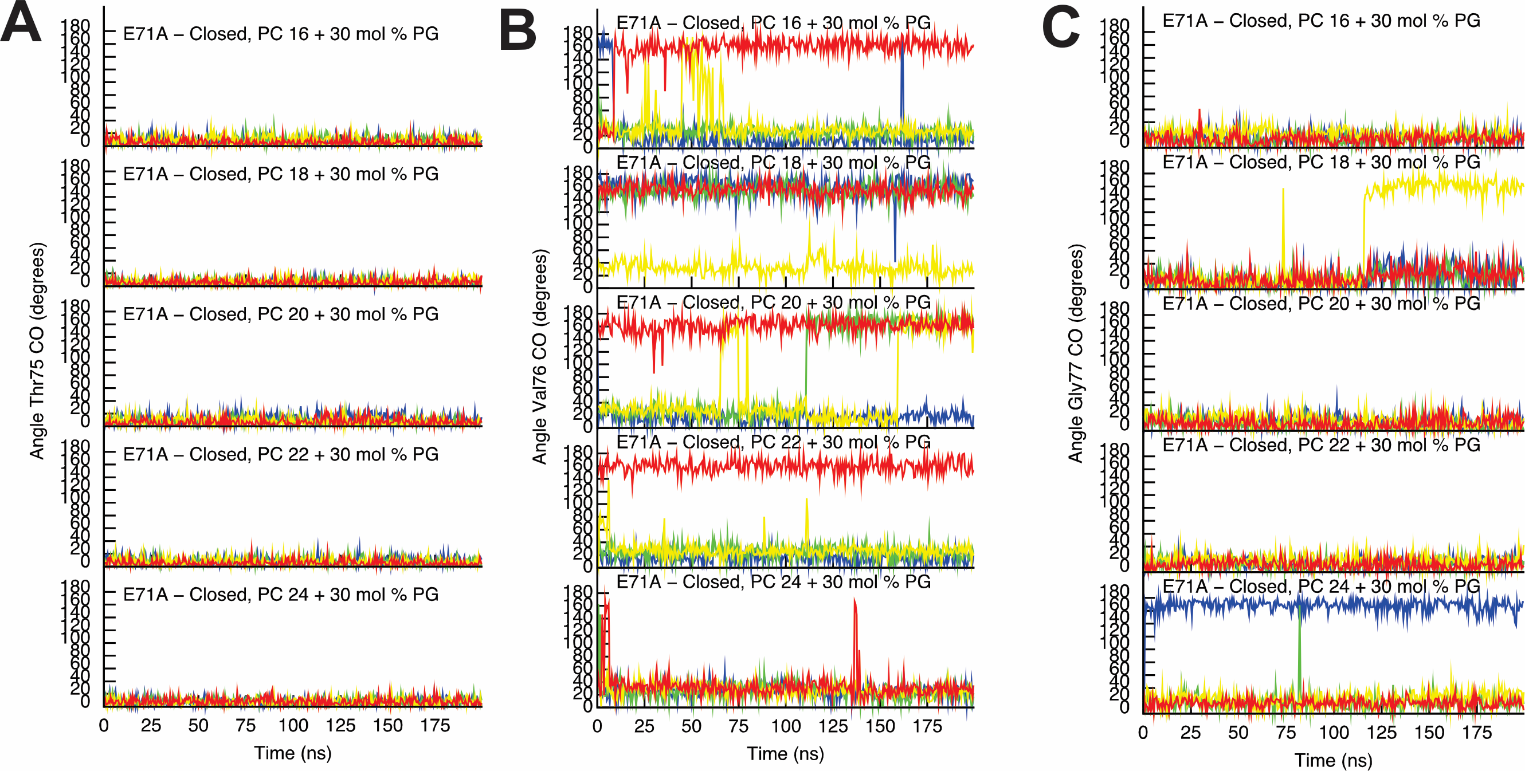
**

**Supplemental Figure S8.** Time-course of the projection of the vector made by the carbonyl carbon and carbonyl oxygen onto the vector made by the carbonyl carbon with the center of the selectivity filter for **(A)** threonine 75 **(B)** valine 76, or **(C)** glycine 77 for closed E71A channels.
